# Nitrate and Ammonium Contribute to the Distinct Nitrogen Metabolism of *Populus simonii* during Moderate Salt Stress

**DOI:** 10.1371/journal.pone.0150354

**Published:** 2016-03-07

**Authors:** Sen Meng, Li Su, Yiming Li, Yinjuan Wang, Chunxia Zhang, Zhong Zhao

**Affiliations:** 1 Key Laboratory of Environment and Ecology in Western China of Ministry of Education, College of Forestry, Northwest A&F University, Yangling, 712100, Shaanxi, People’s Republic of China; 2 State Key Laboratory of Soil Erosion and Dryland Farming on the Loess Plateau, Institute of Soil and Water Conservation, Northwest A&F University, 712100, Yangling, China; Youngstown State University, UNITED STATES

## Abstract

Soil salinity is a major abiotic stressor affecting plant growth. Salinity affects nitrification and ammonification in the soil, however, limited information is available on the influence of different N sources on N metabolism during salt stress. To understand the N metabolism changes in response to different N sources during moderate salt stress, we investigated N uptake, assimilation and the transcript abundance of associated genes in *Populus simonii* seedlings treated with moderate salt stress (75mM NaCl) under hydroponic culture conditions with nitrate (NO_3_^-^) or ammonium (NH_4_^+^). Salt stress negatively affected plant growth in both NH_4_^+^-fed and NO_3_^-^-fed plants. Both NH_4_^+^ uptake and the total N concentration were significantly increased in the roots of the NH_4_^+^-fed plants during salt stress. However, the NO_3_^-^ uptake and nitrate reductase (NR) and nitrite reductase (NiR) activity primarily depended on the NO_3_^-^ supply and was not influenced by salt stress. Salt stress decreased glutamine synthetase (GS) and glutamate synthase (GOGAT) activity in the roots and leaves. Most genes associated with NO_3_^-^uptake, reduction and N metabolism were down-regulated or remained unchanged; while two NH_4_^+^ transporter genes closely associated with NH_4_^+^ uptake (*AMT1;2* and *AMT1;6*) were up-regulated in response to salt stress in the NH_4_^+^-fed plants. The accumulation of different amino acid compounds was observed in the NH_4_^+^- and NO_3_^-^- fed plants during salt treatment. The results suggested that N metabolism in *P*. *simonii* plants exposed to salt enhanced salt resistance in the plants that were fed with NO_3_^-^ instead of NH_4_^+^ as the sole N source.

## Introduction

Salinity is a major abiotic stressor that affects plant growth and productivity [1]. Salt stress constitutes an agricultural and environmental problem worldwide, and salinity is expected to cause serious salinization problems for more than 50% of all arable lands until 2050[2,3]. NaCl stress leads to various deleterious effects at morphological, physiological, biochemical and molecular levels. Salinity affects nitrification and ammonification in the soil [4], thus, the deleterious effect of NaCl stress on plant growth might relate to changes of nitrogen (N) uptake and metabolism [5] since N is one of the key mineral nutrients for plant growth and development.

Different N metabolism has been reported in different species in response to N nutrition and salinity [6,7]. The salt stress tolerance of some durum wheat cultivars (*Triticum turgidum* subsp.*durum*) was dependent on nitrogen availability [7]. Earlier studies also demonstrated that salt stress led to altered ion balance and nitrogen metabolism in rice (*Oryza sativa* L.)[8]. A sufficient N supply compensates and corrects nutritional imbalances in salt-stressed plants. For instance, NH_4_^+^ application could lower the uptake of cations in maize (*Zea mays* L.) [9]. Previous studies have focused on the influence of the N supply on plant growth and antioxidant enzyme activity during salt stress. For example, the growth inhibition of *Catharanthus roseus* under salt stress could largely be ameliorated by fertilization with both ammonium (NH_4_^+^) and nitrate (NO_3_^-^) [10]. NH_4_^+^-fed plants of the halophyte *Spartina alterniflora* exhibited enhanced growth compared to NO_3_^-^-fed plants in high salinity conditions, reflecting the high antioxidant enzyme activity in NH_4_^+^-fed plants [11]. However, different results were obtained in *Pisum sativum*[12] and *Triticum aestivum*[13], suggesting that NH_4_^+^ might increase sensitivity to NaCl treatment. These results suggest that either ammonium or nitrate could improve salt tolerance, depending on the characteristics of the species and its specific ecological conditions.

N metabolism involves the uptake, transport, assimilation and utilization of N for amino acid biosynthesis and ultimately growth [14]. NH_4_^+^ and NO_3_^-^ in soil solution are the two major inorganic N forms for plant. NO_3_^-^ is converted to NH_4_^+^ by NR and NiR. After direct uptake or conversion from NO_3_^-^, NH_4_^+^ is assimilated to glutamine and glutamate via glutamine synthetase (GS) and glutamate synthase (GOGAT), and the products of the GS/GOGAT pathway are required for the biosynthesis of other nitrogenous compounds [14]. Each of these steps might be differently regulated by the N source, leading to differences in N metabolism and the performance of plants during salt stress. N source availability may be an important determinant of species distribution in ecosystems and these differences in the patterns of N-utilization may be a factor in niche separation among species [15–18]. However, limited information is available on the influence of different N sources, such as NO_3_^-^or NH_4_^+^, on N metabolism during salt stress.

The N balance ofNH_4_^+^-fed *Populus canescens* plants was much more affected by salt stress compared to plants supplied with NO_3_^-^which might be due to enhanced proteolysis in NH_4_^+^-fed plants [19]. In pea plants with NH_4_^+^ or NO_3_^-^ under salt stress, the root growth and belowground total N concentration were reduced, and the amino acid concentration increased at the expense of the protein content [12]. NH_4_^+^ might also increase sensitivity to NaCl treatment in *P*. *canescens*; It appears that the reduction of N uptake in plants grown in the solutions containing ammonium as sole N source can be attributed to an increased Na: NH_4_^+^ ratio and competitive inhibition of Na^+^ and NH_4_^+^ [19]. These results suggest that during salt stress, the N source influences N metabolism, including N uptake, assimilation and accumulation (total N, proteins and amino acids), and these effects varied in different species.

*P*. *simonii* is widely distributed in the northern areas of China, and this plant has been recognized as an important afforestation species. In some areas, particularly the Loess Plateau, the soil typically suffers from salt stress [20]. In the present study, to understand the growth and N metabolism of *P*. *simonii* in response to different N sources (NO_3_^-^ or NH_4_^+^) during moderate salt stress, we examined several morphological (root characteristics), physiological (e.g., photosynthesis, N metabolism enzyme activities, total N concentration and amino acid compounds) and molecular (transcript levels of representative genes involved in N metabolism) changes relevant for N metabolism in response to the N source under salt stress. Based on our previous study [5] and the ecological conditions of *P*. *simonii*, we proposed that NH_4_^+^-fed *P*. *simonii* is more susceptible to salt treatment compared to NO_3_^-^-fed plants.

## Materials and Methods

### Plant growth conditions and treatments

*P*. *simonii* cuttings (ca. 15 cm in length, 2 cm in diameter) were obtained from 2-year-old stems and rooted in pots (10 L) filled with fine sand. The cuttings were provided by a tree nursery (Yang Ling, Shannxi, China) and the *P*. *simonii* seedlings were collected within a privately owned forest area with permission given by local forest owners in March 2012. The plants were cultivated in a greenhouse (natural light, day/night 25/20°C, 75% relative humidity). Only one sprout was left for each plant when the bud sprouts had grown to approximately 5 cm. After 4 weeks, similar saplings (ca. 20 cm) were selected, and the roots were carefully washed with tap water. The washed plants were cultivated and acclimated under hydroponic conditions in modified Hoagland’s solution [21] (10 μM EDTA·FeNa, 5 μM MnSO_4_·H_2_O, 1 μM ZnSO_4_·7H_2_O, 1 μM CuSO_4_·5H_2_O, 30 μM H_3_BO_3_, 0.5 μM H_2_MoO_4_, 1 mM KH_2_PO_4_, 1 mM MgSO_4_·7H_2_O, 1 mM CaCl_2_ and 1 mM Na_2_SO_4_, pH 5.5) supplemented with 1 mM NH_4_Cl or KNO_3_ as the N source. A total of 36 plants were treated with salt via the addition of NaCl to the modified Hoagland’s nutrient solution as described above, and the 36 saplings grown in the nutrient solution without NaCl served as the control. For the NaCl treatment, the NaCl concentration was slowly increased to 75mM which is a moderate concentration to study salt stress and avoid early lethal damage (i.e., 25 mM NaCl on the first day, 50 mM on the third day and 75 mM on the fifth day). During this time, aerated nutrient solutions were renewed every other day. The treatment under hydroponic cultivation was maintained for 2 weeks prior to harvest. For each time point and treatment (NO_3_^-^/NH_4_^+^, with and without salt), six plants were cultivated. The plants were harvested at 13:00h at three time points (time point 0 was before the salt application and time points 2 and 3 were 1 and 2 weeks after the start of the salt treatment, respectively).

### Measurement of growth parameters

The gas exchange of three mature leaves (leaf plastochron index = 8–10) was determined for each plant. The net photosynthetic rate was measured from 9:00 to 11:00 h using a portable photosynthesis system (Li-Cor-6400; Li-Cor, Inc., Lincoln, NE) with an attached LED light source (1000 μmol photon m^-2^s^-1^). The CO_2_ concentration in the chambers was 400 μmol mol^-1^ and the air flow was 500 μmol s^-1^. The chlorophyll content of each plant selected was measured using a portable meter (Minolta SPAD 502 Meter). In addition, the height of the main shoot of each plant was measured using a ruler.

The harvested roots and leaves were wrapped in tinfoil and immediately frozen in liquid N. Subsequently, the samples were ground into a fine powder in liquid N using a mortar and pestle and stored at -80°C. The total root lengh, root surface and root volume of each plant were scanned and analyzed using a WinRHIZO root analyzer system (WinRHIZO version 2007b, Regent Instruments Canada, Montreal, Canada).

### Determination of N uptake using the ^15^N tracer method

Prior to harvesting, 3 seedlings of each treatment were treated in a solution containing either ^15^NH_4_^+^ or ^15^NO_3_^-^(3 seedlings for^15^NH_4_Cland 3 seedlings for K^15^NO_3_ because the source of tracer ^15^N recovered in the roots could not be determined when both forms of inorganic N were present in the solution). The roots were rinsed for 10 min in solutions of 1 mM KCl to remove the enriched ^15^N tracer solution that had adsorbed onto the surface of the roots. The roots were subsequently dried for 72 h at 80°C in an oven and homogenized using a mortar and pestle.

The samples were analyzed for ^15^N using a Europa ANCA-SL elemental analyzer coupled to a GVI IsoPrime isotope ratio mass spectrometer (IRMS) at the Chinese Academy of Forestry. Continuous flow analysis was also used to determine the N content of each sample.

### Determination of enzyme activities involved in N assimilation

The nitrate reductase (NR) activity in the tissues was assayed according to the method of Hogberg and Susan [22]. Approximately 0.5 g of the frozen material was ground into a fine powder in an ice bath. The powder was extracted in 4 ml of ice-cold extraction buffer containing 25 mM phosphate buffer (pH 7.5, a mixture of K_2_HPO_4_ and KH_2_PO_4_), 5 mM cysteine and 5 mM EDTA-Na_2_. The extract was centrifuged at 4,000 rpm for 15 min at 4°C, and 0.4 ml of enzyme extract was added to 1.6 ml of the assay mixture (1.2 ml of 0.1 M KNO3-phosphate buffer and 0.4 ml of 2.0 mg ml^−1^ NADH) and incubated at 25°C for 30 min. For the control tube, 0.4 ml of phosphate buffer was used instead of 0.4 ml of NADH. The nitrite (NO_2_^-^) concentration in the buffer was determined after adding 1 ml each of 1% (w/v) sulfanilamide in 3 *N* HCl and 0.02% N-naphthylethylenediamine in water. After incubating for 15 min, all of the samples were centrifuged for 5 min at 4,000 rpm, and the supernatant was read on a spectrophotometer at 540 nm. The NO_2_^-^ concentration was calculated using a standard curve of known NO_2_^-^ concentrations.

The nitrite reductase (NiR) activity was measured as a reduction in the amount of NO_2_^-^in the reaction mixture. The reaction mixture contained 0.1 M potassium phosphate buffer (pH 6.8), 0.4 mM NaNO_2_, 2.3 mM methyl viologen, enzyme extract and 4.3 mM sodium dithionite in 100 mM NaHCO_3_; this was used to initiate the reaction. The reaction was incubated for 30 min at 27°C and was stopped after vortexing and boiling for 1 min. The concentration of NO_2_^-^ remaining in the reaction mixture was determined at 540 nm after reaction with SA and NEDD as described above using a standard curve of known NaNO_2_ concentrations. One unit of NiR activity is defined as 1 mM NO_2_^-^ reduced mg^-1^ protein h^−1^.

For the glutamine synthetase (GS) activity assay, the frozen tissues (approximately 1 g) were ground into 3 ml of 100 mM Tris-HCI (pH 7.6) extraction buffer containing 1 mM EDTA, 1 mM MgCl_2_·6H_2_O and 10 mM 2-mercaptoethanol using an ice-cold mortar and pestle. The homogenate was clarified by centrifugation at 13,000 rpm for 25 min, and 1.2 ml of the crude enzyme extract was added to 1.6 ml of assay mixture containing 0.6 ml of imidazole-muriatic acid buffer (0.25 M, pH 7. 0), 0.4 ml of glutamic acid-Na (0.30 M, pH 7.0), 0.4 ml of ATP-Na (30 M, pH 7.0) and 0.2 ml of MgSO_4_ (0.5 M). The mixture was incubated for 5 min at 25°C. Subsequently, 0.2 ml of hydroxylamine hydrochloride (a 1:1 mixture of 1 M hydroxylamine hydrochloride and 1 M HCl) was added, and the reaction was incubated for 15 min. The reaction was stopped after adding 0.8 ml of acidic FeCl_3_ (2% (W/V) in TCA and 3.5% (W/V) FeCl_3_ in 2% HCl). The samples were centrifuged at 4,000 rpm for 15 min, and the absorbance of the supernatant was measured at 540 nm. The amount of γ- glutamylhydroxamate formed was determined through a comparison with a standard curve that was generated after measuring authentic glutamylhydroxamate in the presence of all assay components. One unit of GS activity was determined as the amount of enzyme required to catalyze the formation of l μM γ-glutamylhydroxamate/min under the present conditions.

Glutamate synthase (GOGAT) activity was measured according to the methods of Singh and Srivastava [23]. The control and treated root and shoot samples (100 mg) were homogenized in 0.2 M sodium phosphate buffer (pH 7.5) containing 2 mM EDTA, 50 mM KCl, 0.1% (v/v) mercaptoethanol and 0.5% (v/v) Triton X-100. The homogenate was centrifuged (at 6,000 *g*) for 15 min at 4°C. The obtained supernatant was used to estimate the GOGAT activity. The reaction mixture (3 ml) included 25 mM sodium phosphate buffer (pH 7.3), which contained 1 mM EDTA, 20 mM l-glutamine, 5 mM 2-oxoglutarate, 100 mM KCl, 1 mM NADH and 0.3 ml of enzyme extract. The absorbance was read at 340 nm for 5 min.

### Analysis of the transcript levels of representative genes involved in N assimilation

Based on previous studies [2,19,24,25], essential members of transporter families for NH_4_^+^ (*AMT1;2* and *AMT1;6*) and NO_3_^-^ (*NRT1;1*, *NRT2;4a*) and genes encoding N assimilation (*NR*, *NiR*, *GS1;3*, *GS2*, *Fd-GOGAT and NADH-GOGAT*) were selected for the transcript analysis by quantitative RT-PCR (qPCR). Total RNA was isolated from the tissues and purified using a plant RNA extraction kit (R6827, Omega Bio-Tek, GA, USA), and trace genomic DNA was digested with DNase I (E1091, Omega Bio-Tek). Aliquots of 1 μg of total RNA were used for first-strand cDNA synthesis using the PrimeScript RT reagent kit (DRR037S, Takara, Dalian, China) in a 20-μl reaction according to the manufacturer’s instructions. PCR was performed in a 20-μl reaction including 10 μl 2× SYBR Green Premix Ex Taq II, 2 μl of cDNA and 1 μl of 20 mM primers (S1 Table) using a LightCycler 96 System (Roche). Actin2/7 was used as a reference gene [26]. Three biological replicates, each with three technical replicates, were assayed for each sample. The reference gene was included on each plate. The efficiencies of all of the PCR reactions were between 95 and 105% (S1 Table).

### Analysis of the amino acid compounds

Soluble amino acid compounds were extracted and analyzed as previously described [2,27]. Approximately 100 mg of the homogenized samples (fine roots, leaves) were incubated in 1 ml of methanol:chloroform (7:3, v/v) and 0.2 ml of HEPES buffer [5 mM ethylene glycol tetraacetic acid (EGTA), 20 mM HEPES, 10 mM NaF, pH 7.0]. The homogenates were incubated on ice for 30 min and subsequently extracted twice with 0.6 ml of distilled water. The aqueous phases were freeze-dried and dissolved in 1 ml of 0.2 M lithium citrate buffer (pH 2.2). The amino compounds were detected using an automated amino acid analyzer (L-8900, Hitachi High-Technologies Corporation, Japan).

### Data processing and statistical analysis

All of the statistical tests were performed using SPSS software (version 20.0, SPSS Inc., Chicago, IL, USA). A three-way ANOVA was used to examine the effects of salt, N treatment and time on the experimental variables. The data were tested for normality prior to further analyses. Differences between the means were determined on the basis of least significant differences (*P* = 0.05).

## Results

### Growth parameters

After 2 weeks of exposure to 75 mM NaCl, the plants exhibited growth inhibition (Table 1).Total root length and stem height were significantly reduced after salt treatment, but no effect of N nutrition was observed (Table 1). Total surface area, chlorophyll content and photosynthesis were also significantly reduced after salt treatment (from T0 until T2 in Table 1). The reductions of chlorophyll content and photosynthesis were much more affected by salt treatment in the NH_4_^+^-fed plants compared to the NO_3_^-^-fed plants (Table 1).

**Table 1:** Growth parameters and photosynthesis of *P*. *simonii* as affected by salt treatment and nitrogen nutrition.

Treatment	Root biomass (g DW)	Total root length (m)	Total root surface area (cm^2^)	Total root volume (cm^3^)	Stem height (cm)	Chlorophyll content (SPAD)	Net photosynthetic rate (mmol CO_2_ m^-2^ s^-1^)
Time point 0	3.14±0.23a	9.74±0.61a	34.96±1.54c	7.53±0.34bc	26.32±0.92d	29.74±0.68a	9.47±0.31a
Time point 1							
NH_4_^+^ without salt	3.36±0.25a	9.45±0.33ab	37.48±2.43bc	8.24±0.35abc	32.0±1.05bc	32.12±0.97a	9.89±0.39a
NH_4_^+^ with salt	3.27±0.27a	8.41±0.39ab	21.61±1.19d	6.81±0.24c	28.5±1.26cd	21.22±1.00b	4.67±0.27c
NO_3_^-^ without salt	3.51±0.17a	9.50±0.45ab	40.39±3.42abc	9.35±0.51ab	33.3±1.26abc	30.78±0.83a	9.29±0.56a
NO_3_^-^ with salt	3.15±0.29a	9.07±0.50ab	24.09±1.47d	7.13±0.86c	28.9±0.69cd	24.22±0.98b	6.66±0.33b
Time point 2							
NH_4_^+^ without salt	3.74±0.23a	10.59±0.87a	44.62±1.68ab	8.05±0.51abc	38.0±0.95a	29.28±1.47a	9.54±0.40a
NH_4_^+^ with salt	3.05±0.22a	7.10±0.17b	17.22±0.84d	6.61±0.22c	29.0±0.99cd	10.60±0.46d	3.85±0.15c
NO_3_^-^ without salt	3.85±0.19a	9.76±0.64a	47.36±1.74a	9.56±0.23a	36.8±1.52ab	30.65±1.44a	9.78±0.42a
NO_3_^-^ with salt	3.29±0.19a	8.07±0.34ab	21.86±0.60d	7.24±0.20c	30.8±1.30cd	15.07±0.47c	6.89±0.18b
P-values N	ns	ns	*	*	ns	*	***
S	ns	***	***	***	***	***	***
T	ns	ns	**	ns	***	***	***
N*S	ns	ns	Ns	ns	ns	*	***
N*T	ns	ns	Ns	ns	ns	ns	**
S*T	ns	**	***	**	***	***	***
N*S*T	ns	ns	ns	ns	ns	ns	**

Data indicate mean ± SE (n = 6). Different letters in the same column indicate significant difference. N, N nutrition; S, salt stress; N*S, interaction of N nutrition and salt treatment.

*P< 0.05

**P <0.01

*** P <0.001; ns, not significant.

### Total N concentration and ^15^N content in the roots and leaves

**Fig 1:**
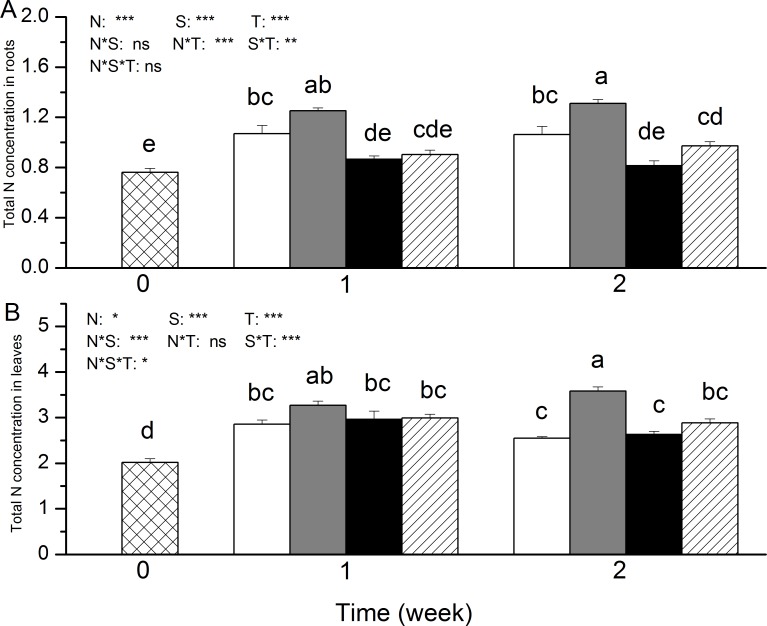
**Total N concentration (g N g^-1^ DW) in roots (A) and leaves (B) of *P. simonii* as affected by salt treatment and nitrogen nutrition.** White boxes indicate ammonium only; grey boxes indicate ammonium with salt; black boxes indicate nitrate only, and striped boxes indicate nitrate with salt. Data indicate mean ± SE (n = 6). Different letters in the same column indicate significant difference. N, N nutrition; S, salt stress; N*S, interaction of N nutrition and salt treatment. *P< 0.05; **P <0.01; *** P <0.001; ns, not significant.

**Fig 2:**
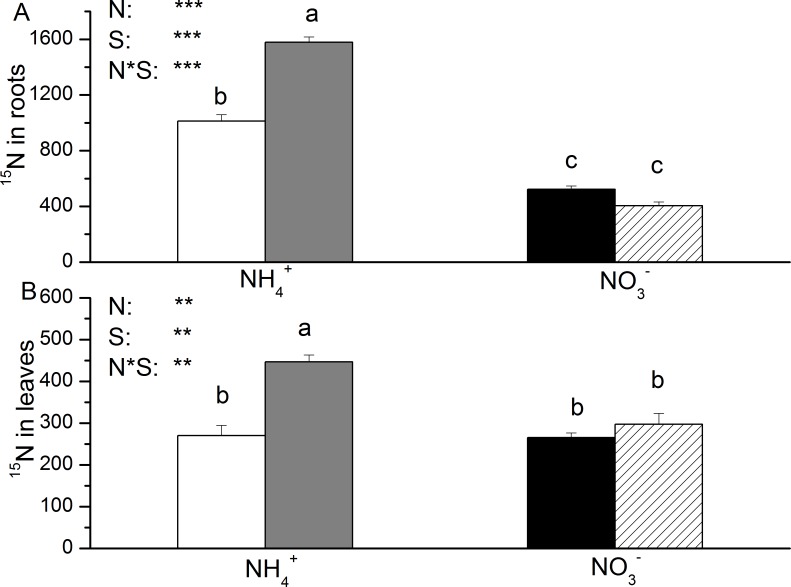
**δ ^15^N (‰) in roots (A) and leaves (B) of *P. simonii* as affected by salt treatment and nitrogen nutrition.** White boxes indicate ammonium only; grey boxes indicate ammonium with salt; black boxes indicate nitrate only, and striped boxes indicate nitrate with salt. Data indicate mean ± SE (n = 3). Different letters in the same column indicate significant difference. N, N nutrition; S, salt stress; N*S, interaction of N nutrition and salt treatment. *P< 0.05; **P <0.01; *** P <0.001; ns, not significant.

Salt treatment significantly increased the total N concentration in the roots of NH_4_^+^-fed plants, particularly after 2 weeks of salt application (Fig 1). In leaves, the increase in total N concentration after salt exposure was significant in the NH_4_^+^-fed plants but not in the NO_3_^-^-fed plants after two weeks (Fig 1).The increase in total N concentration because of salt exposure in the leaves was significantly dependent on N nutrition. Similar to the total N concentration, the ^15^N content in the roots and leaves was significantly increased by salt stress in the NH_4_^+^-fed plants but not in the NO_3_^-^-fed plants (Fig 2). After 2 weeks of salt application, the ^15^N content in roots was greater with NH_4_^+^ compared to NO_3_^-^(Fig 2).

### Enzymes for NO_3_^-^ utilization

**Fig 3:**
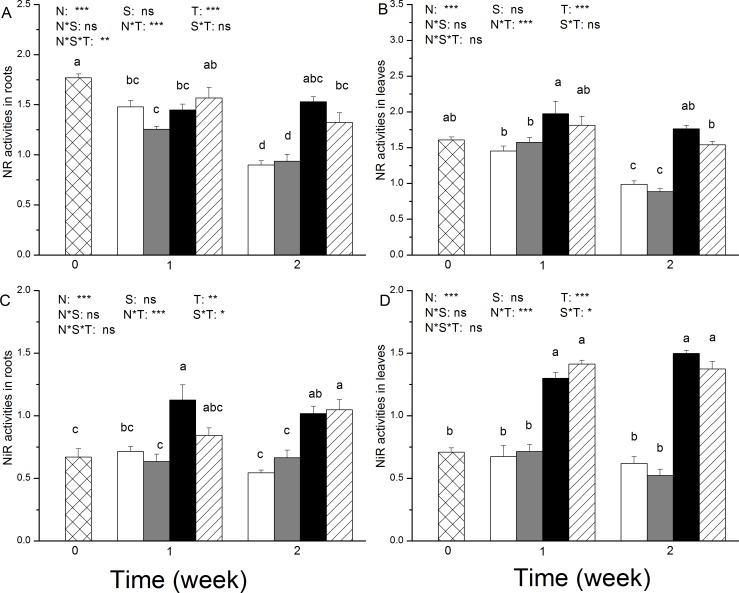
**Activities of nitrate reductase (NR, μM NO3 g^–1^FW h^-1^) (A and B) and nitrite reductase (NiR, mmol NO2- h^-1^ mg^-1^protein) (C and D) in roots and leaves of *P. simonii* as affected by salt treatment and nitrogen nutrition.** White boxes indicate ammonium only; grey boxes indicate ammonium with salt; black boxes indicate nitrate only, and striped boxes indicate nitrate with salt. Data indicate mean ± SE (n = 6). Different letters in the same column indicate significant difference. N, N nutrition; S, salt stress; N*S, interaction of N nutrition and salt treatment. *P< 0.05; **P <0.01; *** P <0.001; ns, not significant.

Significantly higher NR activity was detected in both the roots and leaves of the NO_3_^-^-fed plants compared to the plants that were fed with NH_4_^+^after two weeks of salt treatment (Fig 3). After 2 weeks of salt application, the average NR activity in the roots was 0.9 ± 0.1 μM NO_3_ g^–1^FW h^-1^ (in the control plants) and 0.94 ± 0.17 μM NO_3_ g^–1^FW h^-1^ (in the salt-treated plants) for the NH_4_^+^-fed plants, and it was 1.53 ± 0.12μM NO_3_ g^–1^FW h^-1^ (in the control plants) and 1.33 ± 0.23 μM NO_3_ g^–1^FW h^-1^ (in the salt-treatment plants) for the NO_3_^-^-fed plants (Fig 3). Salt treatment did not significantly decrease NR activity in the roots (Fig 3). Consistent differences in root NR activity were observed as a result of different N sources (NO_3_^-^ versus NH_4_^+^); After 2 weeks of salt application, the average NR activity in the leaves was 0.99 ± 0.12 μM NO_3_ g^–1^FW h^-1^ (in the control plants) and 0.89 ± 0.10 μM NO_3_ g^–1^FW h^-1^ (in the salt-treatment plants) for the NH_4_^+^-fed plants, and it was 1.76 ± 0.12 μM NO_3_ g^–1^FW h^-1^ (in the control plants) and 1.54 ± 0.11 μM NO_3_ g^–1^FW h^-1^ (in the salt-treated plants) for the NO_3_^-^-fed plants (Fig 3). The decrease in foliar NR activity because of salt stress was significant in the NO_3_^-^-fed plants but not in the NH_4_^+^-fed plants after 2 weeks (Fig 3).

Similar to the NR activity, the NiR activity in the roots and leaves was significantly higher in the NO_3_^-^-fed plants than in the NH_4_^+^-fed plants (Fig 3). Salt stress had no effect on the NiR activity in the roots and leaves (Fig 3).

### Enzymes for NH_4_^+^ utilization

**Fig 4:**
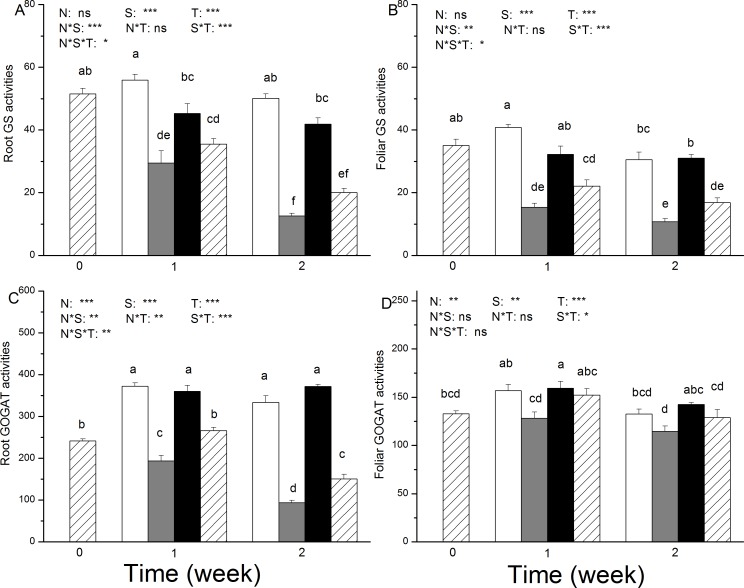
**Activities of glutamine synthetase (GS, U g**^**–1**^**FWh**^**–1**^**) (A and B) and glutamate synthase (GOGAT, nkat g**^**-1**^**protein) (C and D) in roots and leaves of *P*. *simonii* as affected by salt treatment and nitrogen nutrition.** White boxes indicate ammonium only; grey boxes indicate ammonium with salt; black boxes indicate nitrate only, and striped boxes indicate nitrate with salt. Data indicate mean ± SE (n = 6). Different letters in the same column indicate significant difference. N, N nutrition; S, salt stress; N*S, interaction of N nutrition and salt treatment. *P< 0.05; **P <0.01; *** P <0.001; ns, not significant.

The GS activity was significantly decreased upon salt stress in both the roots and leaves, and the amplitude of this effect was dependent on N nutrition (Fig 4). Two weeks of salt treatment resulted in a 75% (in roots) and 65% (in leaves) reduction in GS activity in the NH_4_^+^-fed plants, whereas a 52% (in roots) and 46% (in leaves) reduction was observed in the NO_3_^-^-fed plants (Fig 4).

Salt stress led to a continuous decrease in GOGAT activity in the roots of both the NH_4_^+^ and NO_3_^-^-fed plants during the 2 weeks of salt exposure (Fig 4). This reduction was higher in the NH_4_^+^-fed plants than in the NO_3_^-^-fed plants (Fig 4). However, no changes in GOGAT activity were observed in the leaves after salt exposure (Fig 4).

### Transcriptional regulation of the genes involved in N uptake and metabolism

**Fig 5:**
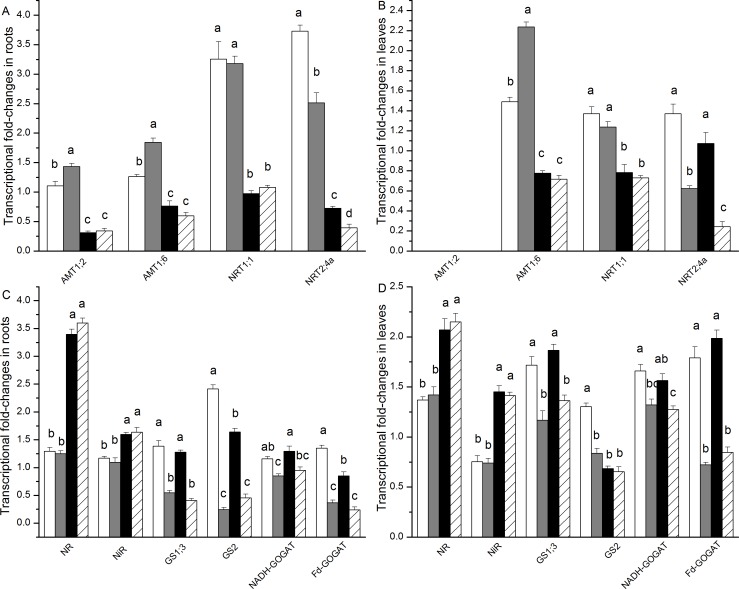
**Transcriptional fold-changes of key genes involved in N uptake and assimilation in roots (A, C) and leaves(B, D) of *P*. *simonii* as affected by salt treatment and nitrogen nutrition.** Signal intensities were calibrated according to a constitutively expressed poplar actin gene (Langer et al. 2004). White boxes indicate ammonium only; grey boxes indicate ammonium with salt; black boxes indicate nitrate only, and striped boxes indicate nitrate with salt. Data indicate mean ± SE (n = 3). Different letters in the same column indicate significant difference.

In the NH_4_^+^-fed plants, the *AMT1;2* and *AMT1;6* transcripts were higher in both the roots and leaves (with the exception of *AMT1;2* in leaves which was specifically expressed in roots) under salt exposure compared to the controls that were not exposed to salt (Fig 5). However, the *AMT1;2* and *AMT1;6* transcript levels did not change as a result of salt treatment when NO_3_^-^ was supplied as the N source (Fig 5). The *NRT1;1* and *NRT2;4a* transcripts in the roots and leaves were higher in the NH_4_^+^-fed plants than in the NO_3_^-^-fed plants (Fig 5). In both the roots and leaves, salt stress suppressed the transcript levels of *NRT2;4a*, whereas no effects on *NRT1;1* expression were observed (Fig 5).

Higher *NR* and *NiR* transcript levels were detected in the roots and leaves of the plants fed with NO_3_^-^ than in the plants fed with NH_4_^+^ (Fig 5). Salt stress did not affect the *NR* and *NiR* transcript levels in the NO_3_^-^ and NH_4_^+^-fed plants (Fig 5).

Substantially lower *GS1;3* and *GS2*transcript levels in the roots and leaves were observed in the salt-treated plants compared to the controls that were not exposed to salt (except in the leaves of the NO_3_^-^-fed plants) (Fig 5). Similarly, salt stress resulted in a significant decrease of the *Fd-GOGAT* and *NADH-GOGAT* transcript levels in both the NO_3_^-^- and NH_4_^+^-fed plants (Fig 5). The genes involved in NH_4_^+^ assimilation (*GS1;3*, *GS2*, *Fd-GOGAT* and *NADH-GOGAT*) were generally not affected by the N source (except *GS2* in both the roots and leaves of the control plants).

### Analysis of the amino compounds in the fine roots and leaves

**Fig 6:**
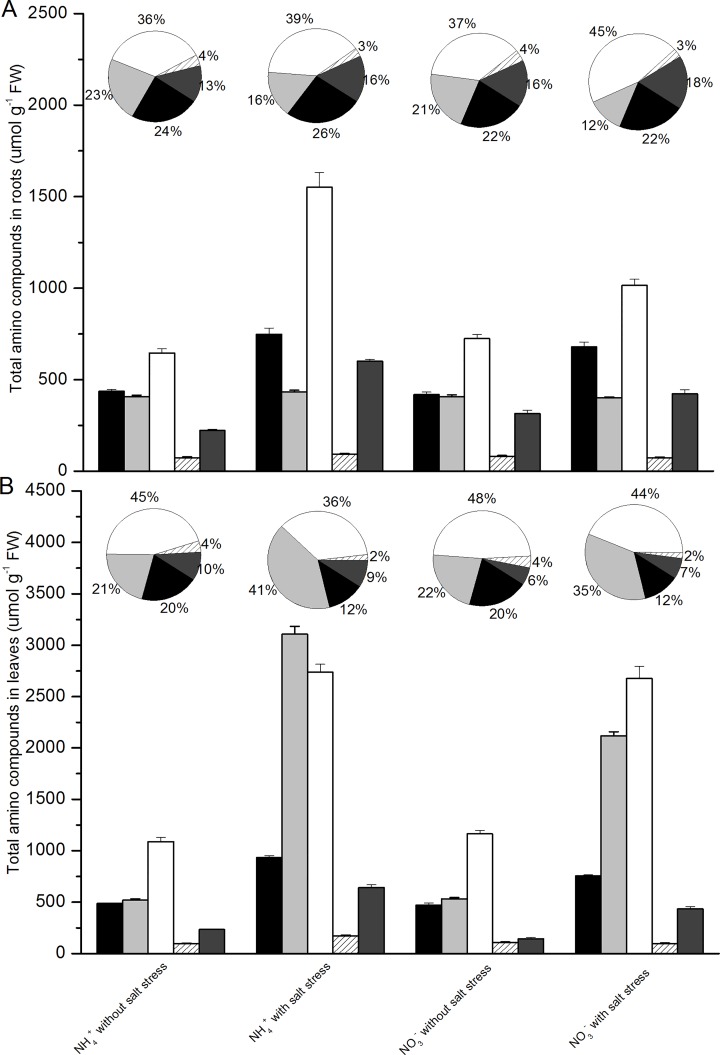
**Amino compounds in roots (A) and leaves (B) of *P. simonii* as affected by salt treatment and nitrogen nutrition.** Amino acids are grouped together deriving from the same pathway. Presented are the percent distributions of these biosynthetic groups, and mean values of sums of amino compounds of each group of three plants and SE. Black: aspartate, threonine, isoleucine, methionine, lysine; light grey: serine, glycine, cysteine; white: glutamate, histidine, arginine, proline; striped: phenylalanine, tyrosine; dark grey: alanine, leucine, valine.

The salt treatment also changed the composition of the amino compounds depending on the N source (Fig 6 and S2 Table). In the roots of the salt-stressed plants grown with NH_4_^+^, the increment of total amino compounds primarily reflected an increase in aspartate-, glutamate- and valine-derived amino compounds (Fig 6A). The main components of the aspartate, glutamate and valine groups were asparagine, glutamine and valine, respectively. Changes in the distribution of the amino compounds were different from the changes in the absolute amounts of amino compound groups. In the roots of the plants grown with NH_4_^+^, the proportions of aspartate (from 24 to 26%), glutamate (from 36 to 39%) and valine (from 13 to 16%) did not significantly change under salt stress (Fig 6A). In the roots of the salt-stressed plants grown with NO_3_^-^, the increase in total amino compounds primarily reflected an increase in aspartate- and glutamate-derived amino compounds (Fig 6A). In the roots of the plants grown with NO_3_^-^, the proportion of the glutamate group increased under salt stress (from 37 to 45%), but the proportion of the serine group decreased (from 21 to 12%), whereas the proportions of the other groups did not change (Fig 6A).

In the leaves of the salt-stressed plants, all groups of amino compounds increased upon salt stress, independent of the N source. The amino acids derived from serine were increased in the NH_4_^+^- (six-fold) and NO_3_^-^-fed plants (four-fold), resulting in an increased contribution to total amino compounds (21 to 41% in the NH_4_^+^-fed plants and from 22 to 35% in the NO_3_^-^-fed plants) (Fig 6B). The main component of the serine group was serine. Amino acids derived from glutamate in both the NH_4_^+^- and NO_3_^-^-fed plants also significantly increased (approximately 2.5-fold) as a result of the salt stress, and the relative proportion of this group of amino compounds slightly decreased (from 45 to 36% in NH_4_^+^-fed plants and from 48 to 44% in NO_3_^-^-fed plants) (Fig 6B). Glutamate was the most abundant amino compound in this group. The absolute amount of glutamate in the leaves of both the NH_4_^+^ and NO_3_^-^-fed plants increased 2-fold upon salt stress. The absolute amount of proline, which also belongs to the glutamate group, increased in the NH_4_^+^-fed plants but did not change in the NO_3_^-^-fed plants in response to salt stress (Fig 6B). The total amount of the aspartate group increased in response to salt stress, but the relative proportion of this group of amino compounds decreased (from 20 to 12% in both the NH_4_^+^- and NO_3_^-^-fed plants)(Fig 6B). The amino compounds of the tyrosine and valine groups increased two- to three-fold as a result of salt stress, independent of the N source, whereas the relative proportions of the two groups did not change (Fig 6B).

## Discussion

Salinity has detrimental effects on plant growth and development, such as reduced root length and changed leaf substructure [28,29]. The effects of salt stress on plant growth in *P*. *simonii* included decreased total root surface area, total volume and photosynthesis inhibition; these effects were dependent on the N source (Table 1). In the present study, the application of salt stress reduced chlorophyll content and net photosynthetic rate more intensively in the NH_4_^+^-fed plants. These results indicate that *P*. *simonii* performs better under salt stress when fed with NO_3_^-^.

The ^15^N uptake and soluble protein in plant materials increased in both the roots and leaves of the NH_4_^+^-fed plants in response to salt stress. However, increases in N uptake and soluble protein were not observed in the NO_3_^-^fed plants. These results indicate enhanced NH_4_^+^ uptake during salt treatment; similar results were also reported in the roots of *Picea glauca* [5,30]. These changes might reflect cation exchange because NH_4_^+^ application could lower the uptake of cations in plant tissues compared to NO_3_^-^-treated plants [9]. In addition, NH_4_^+^ uptake has a lower energy requirement for assimilation in the roots, and the increased NH_4_^+^ concentration could enhance osmotic resistance in rice plants because of aquaporin expression [31]. The accumulation of soluble protein under osmotic resistance has been reported in many species [32–34]. The increased soluble protein might reflect a decrease in amino export [35], the mobilization of storage proteins in tissues [36] and the decomposition and/or the synthesis inhibition of non-soluble proteins. These hypotheses were not investigated in the present study and require further examination.

During assimilation, NO_3_^-^ is converted to NH_4_^+^ by NR and NiR [37]. NH_4_^+^ can be assimilated to glutamine by GS and GOGAT [38,39]. Distinct NO_3_^-^ assimilation has been reported in different species or genotypes in response to salt stress. For instance, previous studies have demonstrated that salt stress decreased NR activity in *Brugueira parviflora* [1], rice (*Oryza sativa* L.) [40] and maize (*Zea mays* L.) [41], whereas in gray poplar (*Populus tremula× alba*), NO_3_^-^ assimilation was not influenced [2]. In the present study, salt stress had only minor effects on NR and NiR activity while the substrate concentration (NO_3_^-^ supply) influences NR and NiR activity in *P*. *simonii*. GS and GOGAT activitywas much lower in the salt-treated plants (except foliar GOGAT activity) compared to the control plants. We suspect that this effect might have two potential explanations: salt stress causes a direct reduction of NH_4_^+^ uptake in the roots in response to the salt stress; and salt stress causes a reduction of NH_4_^+^ production from photorespiration. Na^+^ and Cl^-^ accumulation to toxic levels in rice harmed the chloroplast, influenced photorespiration process, and reduced NH_4_^+^ production from photorespiration, which immediately down-regulated the *OsGS2* and *OsFd- GOGAT* and weakened the GS/GOGAT pathway [8]. However, NH_4_^+^ uptake was not reduced by salt stress. Our results support the second hypothesis.

As an indicator of N uptake and assimilation, the transcript levels of representative genes involved in N uptake and assimilation were analyzed. Several studies have shown that the transcripts of some NH_4_^+^ and NO_3_^-^ transporters (e.g., *AMT1;2*, *AMT1;6*, *NRT1;1* and *NRT2;4a*) play a role in NH_4_^+^ and NO_3_^-^ uptake [5,42,43]. The NH_4_^+^ transporters analyzed in the present study were induced under salt stress in the NH_4_^+^-fed plants but remained unaltered in the NO_3_^-^-fed plants, indicating that the expression of NH_4_^+^ transporters is dependent on the NH_4_^+^ supply and osmotic stress. This idea is consistent with the ^15^N uptake that was observed in the present study and the results of a previous study in which NaCl treatment increased NH_4_^+^ influx and NH_4_^+^ transporters in the roots of *P*. *simonii*[5]. In particular, *AMT1;2* of *P*. *simonii* is only expressed in the roots, which is consistent with previous studies on other poplars [43]. The *NRT1;1* mRNA levels in both the NH_4_^+^- and NO_3_^-^-fed plants were not affected under salt stress. However, the *NRT2;4a* transcripts were lower under salt stress in both the NH_4_^+^-fed and NO_3_^-^-fed plants compared to the controls. The levels of *NRT1;1* and *NRT2;4a* were higher in the NH_4_^+^-fed plants than in the NO_3_^-^-fed plants, irrespective of the presence of salt stress. *NRT1;1* participates in both low- and high-affinity NO_3_^-^ transport and also functions as a NO_3_^-^ sensor to activate the expression of NO_3_^-^-related genes in plants[24,44]. *NRT2;4a* also plays an important role in NO_3_^-^- uptake, particularly under low N conditions. The high levels of *NRT1;1* and *NRT2;4a* transporters in the NH_4_^+^-fed plants suggest that *P*. *simonii* might be sensitive to NO_3_^-^- deficiency. The mRNA level of the *NR* and *NiR* transcripts was not affected under salt stress and was primarily regulated by the substrate concentration (NO_3_^-^), consistent with NR and NiR enzyme activities. However, the transcripts of the genes involved in NH_4_^+^ assimilation were generally decreased in response to salt stress. The down-regulation of the genes involved in NH_4_^+^ assimilation (*GS1*.*3*, *GS2*, *NADH-GOGAT* and *Fd-GOGAT*) might be a harmful response to NaCl toxicity. *P*. *simonii* might accumulate Na^+^ and Cl^-^ to toxic levels under salt stress, and this effect might reduce NH_4_^+^ production from photorespiration, which immediately down-regulates the genes involved in NH_4_^+^ assimilation.

The accumulation of amino acid compounds playing an important role in osmoregulation in response to salt stress has been reported in many plants [24,32–34,45]. The amino compounds were more reactive to salt stress in the NH_4_^+^-fed plants compared to the NO_3_^-^-fed plants. Significant increases in response to salt stress were observed in the serine and glutamate biosynthesis groups. Cysteine (its precursor is serine), glutamate and glycine are important for the synthesis of glutathione (GSH). GSH is important for the stress resistance of plants through the elimination of free radicals. Thus, significant increases of the serine and glutamate biosynthetic groups in response to salt stress might be associated with GSH synthesis. Additionally, aspartate and glutamine were used for the storage and/or transport of N from source to sink tissue [2], indicating intensive N allocation in salt-treated plants.

In summary, salt stress decreased the root development and photosynthesis of *P*. *simonii*, depending on the N source. NH_4_^+^ uptake was enhanced, whereas NH_4_^+^ assimilation was decreased in response to salt stress. NO_3_^-^ metabolism was less affected under salt treatment. The accumulation of amino acid compounds indicates an adaption in response to salt stress. Soils on the Loess Plateau in Northwest China are alkaline, and low levels of N are available. NH_4_^+^ levels are particularly low, making NO_3_^-^ the most available form of N in this region. Thus, *P*. *simonii* growing in these regions exhibits different N uptake and assimilation strategies during salt stress, depending on the N source.

## Supporting Information

S1 Table Specific primers for key genes involved in N uptake and assimilationin *P*.*simonii*. (XLSX)

S2 Table 17 Amino compounds in roots and leaves of *P*.*simonii* as affected by salt treatment and nitrogen nutrition. (XLSX)

S3 Table Total N concentration, activities of nitrate reductase, nitrite reductase, glutamine synthetase and glutamate synthase in roots and leaves of *P*.*simonii* as affected by salt treatment and nitrogen nutrition. (XLSX)

S4 Table Transcriptional fold-changes of key genes involved in N uptake and assimilation in roots and leaves of *P*.*simonii* as affected by salt treatment and nitrogen nutrition. (XLSX)
